# Book Reviews

**Published:** 1896-12

**Authors:** 


### BOOK REVIEWS.

Over the Hookah: The Tales of a Talkative Doctor. By G. Frank Lydston, M.D., Professor of Genito-Urinary Surgery in the Chicago College of Physicians and Surgeons; Professor of Criminal Anthropology in the Kent College of Law, etc. Sold by subscription only. Price, in cloth, gilt top, $4.00. Over 600 hundred pages octavo. Profusely illustrated from the author's designs by C. Everett Johnson. The Fred Klein Publishing Co., Chicago.

Dr. Lydston has written a very interesting book for the busy doctor's diversion. These tales of a talkative (very talkative) doctor are ascribed to a Dr. "William Weymouth" who it appears had had a most varied experience as a practitioner, from the familiar "Doc" of the primitive western mining camp in the sixties to the esteemed family physician in a fashionable quarter of Chicago, and a college professor, of a modern date. The tales were told to a friend of the old doctor's, a medical student who seems to have been a very good listener. The doctor takes occasion, *en passant,* to express his views upon various semi-medical and sociological subjects, and one can easily see that the old gentleman was of broad comprehension and a thorough humanitarian. His reflections on criminal anthropology and the stigmata of degeneracy, in their medico-legal relations, are deserving of wide reading and consideration. The author gets tangled up somewhat on his negro dialect, though he makes an infinitely better success of it than most northern writers do. His "Uncle Abe" may have been originally "fum way down in Georgy," but it is safe to say that his original style of diction bore little resemblance to that here attributed to him. One remark of old Abe's will probably strain the understanding of the average Northern mind. "De ole Jedge . . . treated de niggahs jes' like dey wuz his own chillun. Doan' s'pose you folks up Norf undahstan' nuffin 'bout dat, sah, but dar's many er po' ole niggah dat wish dar nebbar wuz no war!" Some of the stories of

Western life have a distinct Bret Harte flavor, and some of the character sketches that Dr. Weymouth draws "over the hookah" have very striking counterparts in the writings of the latter author.

The book is well bound and printed and nicely illustrated. It will not fail to interest both the medical and non-medical reader.

---

A TREATISE ON SURGERY. By American Authors. Edited by Roswell Park, M.D., Professor of Surgery and Clinical Surgery, Medical Department, University of Buffalo, N. Y. In two very handsome octavo volumes, comprising 1,600 pages, with 786 engravings, largely original, and 37 full-page plates in colors and monochrome. Volume II, Special Surgery. Price per volume, cloth, $4.50; leather, $5.50. Net. Lea Brothers & Co., publishers, Philadelphia and New York.

The second volume of " Park's Surgery by American Authors," deals with the whole domain of Special Surgery, and in connection with the first volume which comprised "General Surgery and Surgical Pathology," it completes this latest exponent of the world's surgical knowledge. Inspection of the list of contributors shows that Professor Park has been able to secure the co-operation of a most representative body of American surgeons, and likewise that he has assigned the subjects with rare discretion to those most competent to write authoritatively on each topic.

The editor, Dr. Park, describes the surgical diseases and injuries of the head in a very complete chapter of seventy pages. Dr. E. H. Bradford, of Harvard, writes of the surgery of the spine; Dr. F. S. Dennis, of New York, of the surgery of the chest; Dr. M. H. Richardson, of Boston, of the abdomen and hernia; Dr. C. B. Kelsey, of New York, of the rectum and sigmoid flexure; Dr. R. W. Lovett, of Boston, of orthopedic surgery; Dr. R. Matas, of New Orleans, of amputations; Dr. Duncan Eve, of Nashville, of the heart and large bloodvessels, etc. The surgical diseases and injuries of the eye, ear, nose and throat are described respectively by Drs. C. S. Bull, C. J. Blake, and D. B. Delavan. Everything of common interest and importance connected with the surgery of hose regions is concisely described. The last chapter (by the editor) is devoted to skiography, or the application of the Roentgen rays to surgery, giving description of the apparatus and the method of using it.

These two volumes of Park's Treatise on Surgery make the most important late contribution to American surgical literature.

---

A TEXT-BOOK OF MATERIA MEDICA, THERAPEUTICS, AND PHARMACOLOGY. By George Frank Butler, Ph.G., M.D., Professor of Materia Medica and Clinical Medicine in the College of Physicians and Surgeons, Chicago; Professor of Materia Medica and Therapeutics, Northwestern University, Woman's Medical School; Attending Physician to Cook County Hospital, etc. W. B. Saunders, 925 Walnut street, Philadelphia. Price, $4 net.

This book, which is dedicated to the medical students of the United States, is well arranged and beautifully printed. A description is given of the various drugs in use, which is sufficient, but not too long for the purpose of bringing salient points before the mind of the student. A section is especially given over to correcting of prescriptions, common errors in Latin being pointed out. The book ends with a clinical index in which the various drugs used in each disease, are given with reference to their description in the text. Dr. Butler is to be congratulated upon a production so complete and useful. A. W. S.

---

PHYSICIAN'S VISITING LIST FOR 1897. P. Blakiston, Son & Co., 1012 Walnut street, Philadelphia.

This popular visiting list has now reached its forty-sixth year of publication, and hardly needs more than mentioning. This one contains a few more pages, and the leather backs are a little thicker than preceding editions. It is a convenient and serviceable visiting list.

---

THE NON-HEREDITY OF INEBRIETY. By Leslie E. Keeley, M.D., Dwight, Illinois. Published by Scott, Foresman & Co., Chicago.

The author endeavors in these pages to set forth in detail his theory that inebriety is a disease that can be readily cured, and that it is not hereditary.

